# Clinical impact of routine second‑opinion reading of [¹⁸F]FDG‑PET/CT in newly diagnosed invasive bladder cancer: A multicentre study in a centralised referral network

**DOI:** 10.1007/s00259-026-08145-1

**Published:** 2026-08-25

**Authors:** Renee A. G. Lijnen, Misha L. Vierling, Joanneke B. Ringia, Kees Hendricksen, Gijs H. KleinJan, Arnoud W. Postema, Wouter V. Vogel, Thierry N. Boellaard, Arthur J. A. T. Braat, Linda Heijmen, Bas W. G. van Rhijn, Laura S. Mertens

**Affiliations:** 1 Department of Urology, Netherlands Cancer Institute, Amsterdam, The Netherlands; 2 Department of Urology, Leiden University Medical Centre, Leiden, The Netherlands; 3 Department of Nuclear Medicine, Netherlands Cancer Institute, Amsterdam, The Netherlands; 4 Department of Radiation Oncology, Netherlands Cancer Institute, Hospital, Amsterdam, The Netherlands; 5 Department of Radiology, Netherlands Cancer Institute, Amsterdam, The Netherlands; 6 Department of Radiology and Nuclear Medicine, UMC Utrecht, Utrecht, The Netherlands; 7 Department of Nuclear Medicine, Leiden University Medical Centre, Leiden, The Netherlands; 8 Department of Urology, Caritas St Josef Medical Centre, University of Regensburg, Regensburg, Germany

**Keywords:** Urinary bladder neoplasms, Fluorodeoxyglucose F18, Positron-emission tomography computed tomography, Second-opinion reading, Interobserver agreement, Lymphatic metastasis

## Abstract

**Purpose:**

[¹⁸F]FDG‑PET/CT is increasingly used to stage invasive bladder cancer (IBC), resulting in growing demand at tertiary centres for time‑intensive second‑opinion reading of externally acquired scans. However, the incremental clinical value of routine reinterpretation is unclear. We evaluated the clinical impact of second‑opinion [¹⁸F]FDG‑PET/CT reading in a centralised referral network in the Netherlands.

**Methods:**

In this multicentre retrospective diagnostic cohort study, consecutive patients with histologically confirmed high-grade IBC who underwent externally acquired [¹⁸F]FDG‑PET/CT followed by expert second‑opinion reading at two tertiary centres were evaluated. Initial staging reports were compared with second-opinion staging assessments. Clinically meaningful discrepancies were defined as changes in nodal status (cN0 vs. cN+) or distant metastatic status (cM0 vs. cM1 or cM1a vs. cM1b). Treatment recommendation changes directly attributable to second-opinion reading were recorded.

**Results:**

Six hundred patients were included. Agreement between initial and second‑opinion reading was high for both nodal and metastatic staging (κ = 0.89 and κ = 0.87, respectively). Clinically meaningful discrepancies occurred in 33 patients (5.5%), resulting in revised treatment recommendations in 22 patients (3.7%). Suspected nodal disease on the initial report was associated with higher odds of discrepancy at second-opinion reading (OR 2.1, 95% CI 1.0–4.4).

**Conclusion:**

In this multicentre cohort, routine second‑opinion reading of externally acquired [¹⁸F]FDG‑PET/CT for IBC yielded limited incremental clinical impact within a centralised referral network in the Netherlands. These findings suggest that a more selective approach to second-opinion reading, particularly for patients with suspected nodal disease on the initial report, may warrant further evaluation.

## Introduction

The role of [¹⁸F]fluorodeoxyglucose positron emission tomography/computed tomography ([¹⁸F]FDG‑PET/CT) in staging invasive bladder cancer (IBC) is expanding. Compared with conventional imaging, [¹⁸F]FDG‑PET/CT can provide additional information on nodal and distant metastases [1–3] and is increasingly incorporated into staging guidelines [4–6]. As bladder cancer care becomes increasingly centralised [7–10], treatment is typically performed in tertiary centres, while imaging is often acquired externally. Consequently, patients are increasingly referred with externally acquired imaging requiring reinterpretation.

For anatomical imaging modalities such as CT and MRI, second‑opinion reading has been shown to affect staging and treatment planning [11, 12]. In contrast, evidence regarding routine reinterpretation of [¹⁸F]FDG-PET/CT is limited and heterogeneous, with reported discrepancy rates from 2% to 19% across several malignancies [13–16]. Many prior studies evaluated selectively requested reinterpretations, potentially overestimating the clinical yield of routine review. Consequently, the incremental value of systematic second-opinion reading of externally acquired [¹⁸F]FDG-PET/CT in routine bladder cancer care remains unclear.

Given limited specialist capacity and rising healthcare costs, the key question is no longer whether reinterpretation can alter staging, but whether it justifies routine use. We therefore conducted a multicentre study evaluating the clinical impact of routine second‑opinion [¹⁸F]FDG-PET/CT interpretation on staging and treatment recommendations in patients with IBC within a centralised bladder cancer network.

## Materials and methods

### Study design and patients

This multicentre retrospective diagnostic cohort study was conducted at two tertiary cancer centres, the Netherlands Cancer Institute (NCI) and Leiden University Medical Centre (LUMC) after institutional review board approval (IRBd25‑199).

Consecutive patients with histologically confirmed high-grade IBC referred with externally acquired [¹⁸F]FDG‑PET/CT for primary staging were included at the NCI (2014–2024). An independent LUMC cohort (2023–2024) was included to assess reproducibility beyond a single institutional workflow. This study is reported in accordance with the STROBE guideline.

### Second-opinion reading and staging assessment

At both centres, externally acquired [¹⁸F]FDG‑PET/CT underwent routine second‑opinion reading as part of multidisciplinary clinical practice. The availability of post‑excretion imaging, which reduces urinary tracer interference in pelvic assessment, was recorded. Scans were re-evaluated by experienced nuclear medicine physicians with access to the initial report, clinical information, and prior imaging.

Clinical tumour (cT), nodal (cN), and metastatic (cM) stages were defined according to the TNM classification (9th edition) [17]. cT stage was derived from CECT and transurethral resection of bladder tumour (TURB). All lesions reported as suspicious for metastatic disease were recorded. Stage classifications from the initial report were compared with those after expert second-opinion reading. Clinically meaningful discrepancies were predefined as: (i) cN0 vs. cN+; (ii) cM0 vs. cM1; (iii) cM1a vs. cM1b. Nodal discrepancies in the presence of distant metastatic disease were not considered clinically meaningful.

### Clinical impact

Cases with clinically meaningful discrepancies were reviewed by a multidisciplinary expert panel (two urologists and a nuclear medicine physician), who assessed whether treatment recommendations changed as a direct consequence of the second-opinion reading, rather than broader multidisciplinary discussion or institutional policy. Treatment recommendations followed EAU guidelines and institutional protocols [6]. Where available, discrepant findings were corroborated using histopathology or unequivocal clinical and/or radiological follow-up, reported descriptively.

### Outcome and statistical analysis

The primary objective was to assess clinical utility rather than diagnostic accuracy of second-opinion reading. Agreement between initial and second-opinion reading was assessed using Cohen’s kappa (κ). Logistic regression identified predictors of clinically meaningful discrepancies, reported as odds ratios (ORs) with 95% confidence intervals (CIs). Multivariable models included a priori defined covariates (cT stage and cN stage on initial report); univariable analyses were exploratory. In a post-hoc analysis, discrepancy rates were compared between earlier (2015–2020) and more recent (2021–2024) NCI patients using Fisher’s exact test. Analyses were performed using SPSS version 30.0 (IBM Corp., Armonk, NY).

## Results

### Patients

Of 669 patients with second‑opinion [¹⁸F]FDG‑PET/CT reading, 69 were excluded (treatment before scan, *n* = 40; urachal cancer, *n* = 17; low‑grade or non‑invasive bladder cancer, *n* = 12), leaving 600 patients eligible for analysis. Patients were referred from 45 hospitals nationwide.

Most patients were male (72%), with a median age of 69 years (interquartile range [IQR] 61–76 years). Muscle‑invasive bladder cancer was present in 93%, and pure urothelial carcinoma was the predominant histology (85%). Post‑excretion [¹⁸F]FDG‑PET/CT imaging was performed in 72%. Median TURB-[¹⁸F]FDG‑PET/CT interval was 14 days (IQR 7–20). Baseline characteristics are shown in Table 1. No data were missing for the variables analysed.

**Table 1:** Patient and tumour characteristics (n 600)

	Total	NCI	LUMC
	*N*	*N*	*N*
Included patients	600	500	100
Sex Female Male	169 (28%) 431 (72%)	135 (27%) 365 (73%)	34 (34%) 66 (66%)
Median age, years (IQR)	69 (61–76)	68 (60–75)	73 (65–77)
Histological subtype Pure UC Squamous differentiation Glandular differentiation Neuroendocrine differentiation Sarcomatoid differentiation Other	510 (85%) 48 (8.0%) 7 (1.2%) 23 (3.8%) 10 (1.7%) 2 (0.3%)	429 (86%) 38 (7.6%) 7 (1.4%) 16 (3.2%) 8 (1.6%) 2 (0.4%)	81 (81%) 10 (10%) - 7 (7%) 2 (2%) -
Clinical tumour stage based on CECT and TURB cT1 cT2 cT3 cT4	43 (7.2%) 221 (37%) 239 (40%) 97 (16%)	40 (8.0%) 176 (35%) 199 (40%) 85 (17%)	3 (3.0%) 45 (45%) 40 (40%) 12 (12%)
Clinical nodal stage on initial [^18^F]FDG-PET/CT report cN0 cN+	434 (72%) 166 (28%)	360 (72%) 140 (28%)	74 (74%) 26 (26%)
Post‑excretion [¹⁸F]FDG‑PET/CT imaging Yes No	434 (72%) 166 (28%)	338 (68%) 162 (32%)	96 (96%) 4 (4.0%)
Median interval between TURB and [^18^F]FDG-PET/CT, days (IQR)	14 (7–20)	14 (7–20)	15 (2–21)

[^**18**^F]FDG-PET/CT= Fluor-18-deoxyglucose positron emission tomography with computed tomography, *NCI* The Netherlands cancer institute, *LUMC* Leiden University Medical Centre, *IQR* interquartile range, *UC* urothelial carcinoma, *CECT* contrast enhanced computed tomography, *TURB* transurethral resection of bladder tumour

### Staging discrepancies

**Fig. 1:**
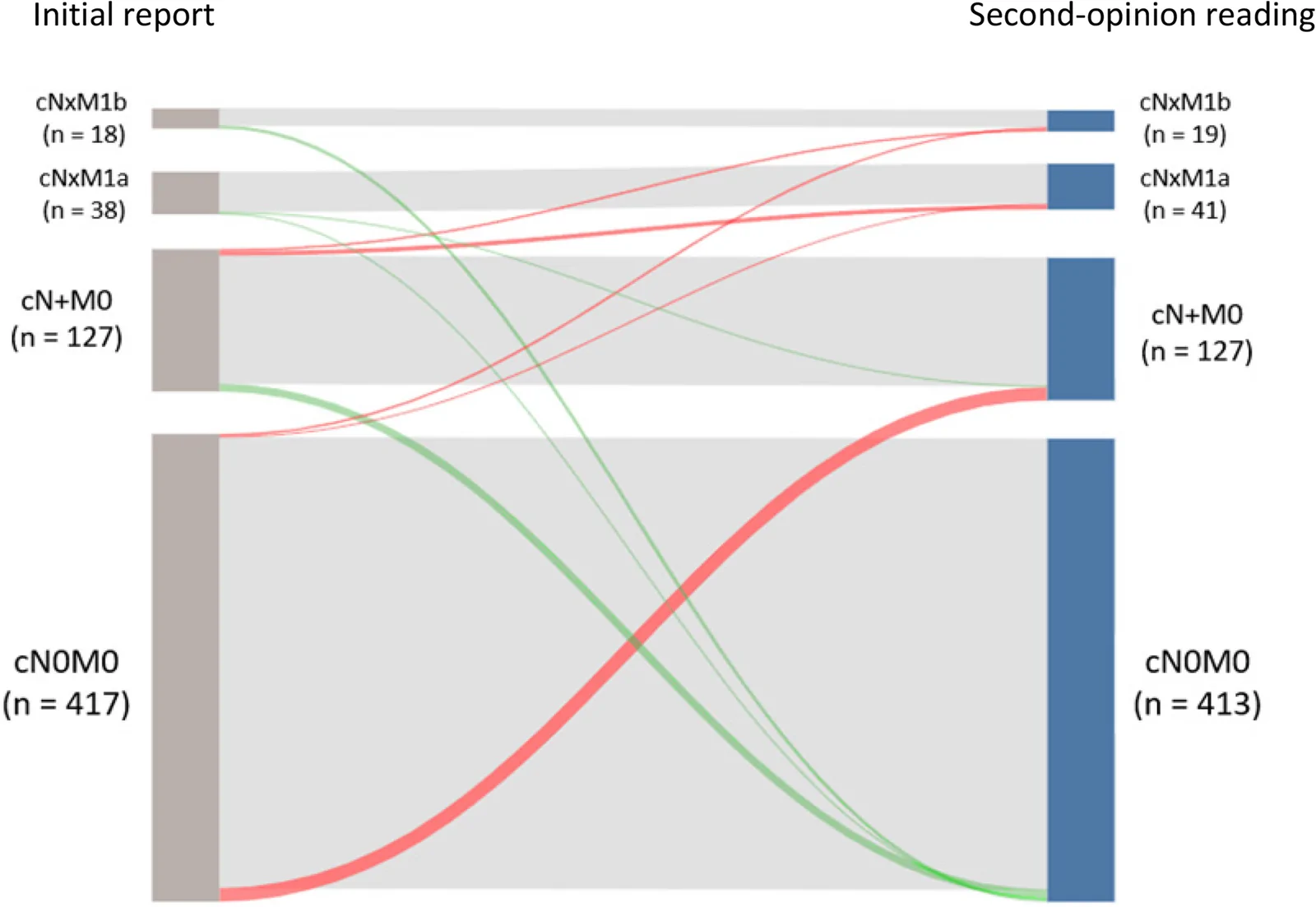
Stage migration between initial report and second-opinion reading of [¹⁸F]FDG-PET/CT (*n* = 600). [^18^F]FDG-PET/CT= Fluor-18-deoxyglucose positron emission tomography with computed tomography. Sankey diagram illustrating stage migration between the initial [¹⁸F]FDG-PET/CT report and second-opinion review in 600 patients with invasive bladder cancer. Most patients showed concordant staging after second-opinion reading. Clinically meaningful stage migration occurred in 33 patients (5.5%), including 21 upstaging events (3.5%; red) and 12 downstaging events (2.0%; green). Flows represent changes in assigned stage category and should not be interpreted as evidence that either interpretation was diagnostically correct

Agreement between initial report and second-opinion reading was high for both nodal and metastatic staging (κ = 0.89 and κ = 0.87, respectively). Overall, 567/600 patients (95%) demonstrated no clinically meaningful stage discrepancies. Clinically meaningful discrepancies were identified in 33 patients (5.5%), with comparable rates at both institutions (5.6% and 5.0%). Upstaging was more frequent than downstaging (3.5% vs. 2.0%; Fig. 1).

**Fig. 2:**
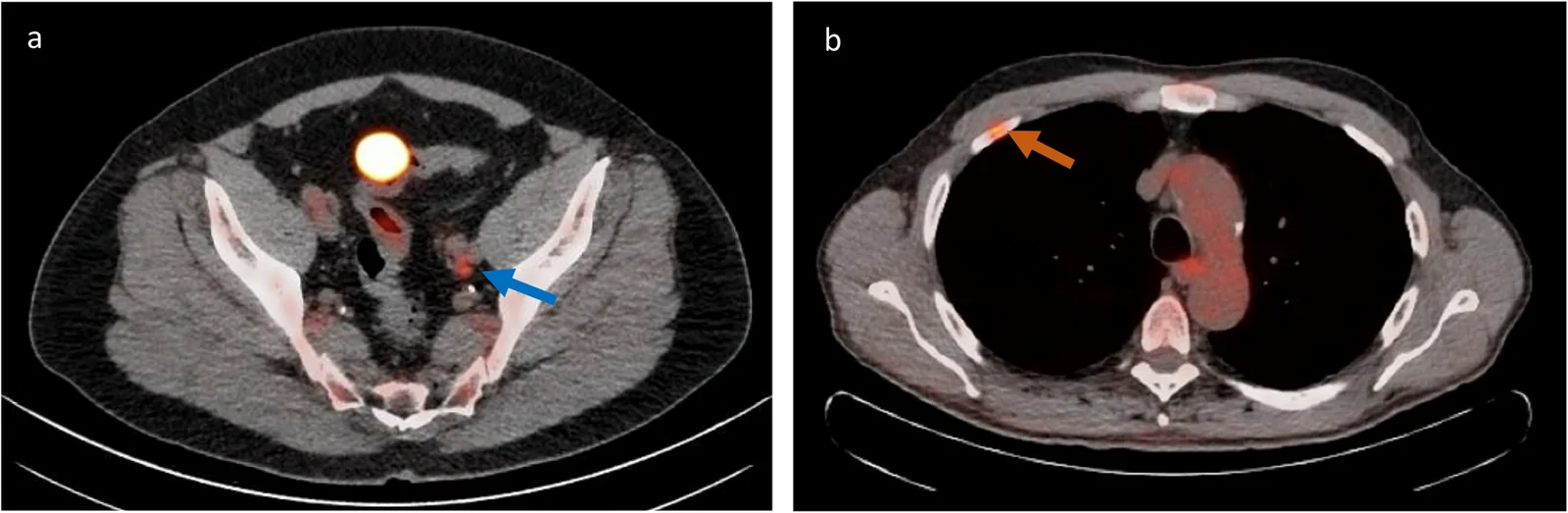
Representative [¹⁸F]FDG-PET/CT scans showing clinically meaningful discrepancies between the initial report and second-opinion reading. **a**. [¹⁸F]FDG-PET/CT of a 62-year-old male with cT1 high-grade bladder cancer demonstrating an FDG-avid lesion in the left obturator region (blue arrow). The lesion was not described in the initial report but was considered suspicious for regional nodal metastasis on second-opinion review, resulting in a revised multidisciplinary treatment recommendation with addition of neoadjuvant therapy. **b**. [¹⁸F]FDG-PET/CT of a 67-year-old male with cT4 bladder cancer showing an FDG-avid lesion in the right third rib (orange arrow). The lesion was initially interpreted as post-traumatic. On second-opinion review, it was considered suspicious for osseous metastasis, resulting in revised staging and a change from curative-intent local therapy to systemic treatment alone. In both cases, subsequent clinical follow-up supported the second-opinion interpretation. These examples are intended solely to illustrate the nature and potential clinical consequences of discrepant interpretations and should not be interpreted as evidence of superior diagnostic performance of second-opinion review

Most clinically meaningful discrepancies involved lymph node assessment (26/33; 79%), including changes in regional nodal stage (cN; 19/26) and extra‑regional nodal stage (cM1a; 7/26). The remaining discrepancies involved non-nodal distant metastases (cM1b; 7/33; 21%), including four upstaging cases (bone, muscle, and peritoneum [*n* = 2]) and three downstaging cases (kidney, muscle, and bone). Discrepancy rates were highest among patients initially staged as cN+M0 (13/127; 10%) or cN+/-M1b (3/18; 17%). Discrepancy rates declined over time in the NCI cohort (8.4% in 2015–2020 vs. 3.3% in 2021–2024; OR 2.68, 95% CI 1.19–6.05, *p* = 0.018). Representative cases are shown in Fig. 2.

### Clinical impact

Among the 33 patients with clinically meaningful staging discrepancies, treatment recommendations changed in 22 patients (67% of discrepant cases; 3.7% of the total cohort). In the remaining 11 (33%), treatment recommendations remained unchanged because advanced local disease already warranted NAT.

Most treatment changes reflected transitions between local treatment and systemic treatment alone following upstaging from cM0 to cM+ disease (9/22). Nodal upstaging prompted initiation of NAT in four patients, while nodal downstaging led to omission in another four. Downstaging of metastatic disease resulted in a shift from systemic therapy alone to local treatment, with or without NAT (5/22). Treatment changes are detailed in Table 2.

**Table 2:** Changes in treatment recommendations of patients with clinically relevant stage discrepancy between initial [^18^F]FDG-PET/CT report and second-opinion reading (n = 33)

		Treatment recommendation based on second-opinion reading			Total	Treatment change
		Local treatment	NAT + local treatment	Systemic treatment alone		
Treatment recommendation based on initial report	Local treatment	0	4	1	5	5 (100%)
	NAT + local treatment	4	11	8	23	12 (52%)
	Systemic treatment alone	1	4	0	5	5 (100%)
	Total (N)	5 (15%)	19 (58%)	9 (27%)	33 (100%)	22 (67%)

*FDG-PET*/*CT* [¹⁸F]-fluorodeoxyglucose positron emission tomography with computed tomography, *NAT* neoadjuvant therapy

Rows represent treatment recommendations based on the initial [¹⁸F]FDG-PET/CT report; columns represent treatment recommendations after second-opinion reading. Among 33 patients with clinically meaningful stage discrepancies, treatment recommendations differed after second-opinion reading in 22 patients, corresponding to 3.7% of the total cohort: Of these 22 treatment changes, 13 followed upstaging and 9 followed downstaging after second-opinion reading

Corroborative evidence, comprising histopathology (*n* = 8) or unequivocal clinical and/or radiological follow-up (*n* = 17), was available for 25/33 discrepant cases and supported the second-opinion interpretation in 20/25.

### Factors associated with clinically meaningful discrepancy

Suspected nodal disease on the initial report was associated with clinically meaningful discrepancies in univariable analysis, remaining significant in multivariable analysis (OR 2.1, 95% CI 1.0–4.4, *p* = 0.041; Table 3). The other variables tested were not associated with discrepancies.

**Table 3:** Factors associated with clinically meaningful stage discrepancy between the initial [¹⁸F]FDG-PET/CT report and second-opinion reading

Parameter	Regression					
	Univariable			Multivariable		
	OR	95% CI	p-value^a^	OR	95% CI	p-value^a^
Sex Female Male Age (per 10-year increase)	Ref 0.67 0.97	0.32–1.4 0.69–1.35	0.28 0.84			
Histological subtype No (conventional UC) Yes	Ref 1.0	0.38–2.7	0.98			
Clinical tumour stage						
cT1-cT2	Ref					
cT3-cT4	1.6	0.77–3.4	0.21	1.5	0.69–3.2	0.31
Nodal stage on initial report						
cN0	Ref					
cN+	2.3	1.1–4.7	0.022	2.1	1.0–4.4	0.041
Interval between TURB and [¹⁸F]FDG-PET/CT in days						
≤ 21	Ref					
> 21^b^	0.67	0.31–1.5	0.33			
[¹⁸F]FDG-PET/CT with post-excretion imaging						
No	Ref					
Yes	0.50	0.24–1.0	0.055			

^a^Wald p-values, ^b^patients in whom TURB was performed after [¹⁸F]FDG-PET/CT or more than 21 days before [¹⁸F]FDG-PET/CT

*OR* odds ratio, *CI* confidence interval, *UC* urothelial carcinoma, *TURB* transurethral resection of the bladder tumour

Logistic regression was used to evaluate clinicoradiological factors associated with clinically meaningful stage discrepancies. Univariable analyses were exploratory and described crude associations. Multivariable models included a priori defined covariates (clinical tumour and nodal stage), with the number of variables limited to avoid overfitting because of limited number of events

## Discussion

In this multicentre diagnostic cohort evaluating routine second-opinion reading of externally acquired [¹⁸F]FDG-PET/CT in IBC, concordance between initial and second-opinion reading was high (κ = 0.87–0.89). Clinically meaningful staging discrepancies occurred in 5.5% of patients, resulting in treatment changes in 3.7%. These findings suggest that the incremental yield of routine second-opinion reading is limited within a centralised referral network in the Netherlands. Rather than questioning the value of expert reinterpretation itself, our results challenge whether systematic reinterpretation of all externally acquired scans is the most efficient approach.

Clinically meaningful discrepancies occurred more frequently in patients with suspected nodal disease on the initial report, likely reflecting the difficulty of distinguishing inflammatory from malignant lymph nodes. Accordingly, targeting second-opinion review to patients with suspected nodal involvement may optimise use of subspecialised imaging expertise; given the limited number of events and borderline confidence interval, this should be considered hypothesis-generating.

Our findings differ from previous studies reporting higher discrepancy rates for second‑opinion [¹⁸F]FDG‑PET/CT reading across different malignancies. However, many studies focused on selectively requested reviews, likely enriching for complex cases and overestimating clinical impact [13, 14, 16]. These studies also largely reflect earlier imaging eras; recent PET/CT advances and implementation experience have since improved image quality and interpretation, contributing to lower discrepancy rates in contemporary practice [18, 19], consistent with the temporal decline observed in our cohort. Moreover, our nationwide, multicentre design may better reflect contemporary real-world staging workflows within centralised cancer networks.

Our findings should be interpreted in light of the study’s design and setting. A key strength is the homogeneous cohort limited to patients undergoing primary staging of IBC. Second-opinion readers had access to the initial report, potentially introducing anchoring bias, although this reflects real-world practice. Second-opinion reading was performed in a multidisciplinary setting across a broad range of referral hospitals, supporting generalisability. This study was designed to evaluate clinical impact rather than diagnostic accuracy; comprehensive reference standard verification was not feasible for all discrepant findings. Where available, corroborative evidence largely supported second-opinion interpretation. These descriptive findings should not be interpreted as assessment of diagnostic accuracy. Finally, findings were generated within a centralised healthcare system with relatively homogeneous nuclear medicine training and expertise, possibly limiting generalisability to more heterogeneous healthcare settings.

Although clinically meaningful discrepancies occurred in a minority of patients, their impact on individual management can be substantial. This may be partly attenuated by Enfortumab Vedotin plus Pembrolizumab as first-line therapy across unresectable locally advanced and metastatic disease, reducing reliance on the nodal/metastatic distinction for treatment choice [20]. This distinction remains relevant for patients with potentially resectable nodal disease, where staging still guides candidacy for multimodal treatment. Our findings argue not against second-opinion reading itself, but question its routine application to all externally acquired scans. The observed association with suspected nodal disease on the initial report suggests that a more selective strategy may warrant further evaluation to better allocate subspecialised imaging expertise in contemporary bladder cancer care.

## Data Availability

The datasets generated during and/or analysed during the current study are available from the corresponding author on reasonable request.
